# Empowerment and Community Process Diagnosis to Promote Epidemiological Surveillance of Nursing Diagnoses: A MAIEC-Based Study in the Autonomous Region of the Azores, Portugal

**DOI:** 10.3390/ijerph23070830

**Published:** 2026-06-24

**Authors:** Pedro Melo, Renata Silva, Flávio Vieira, Susana Barbeitos, Susana Figueiredo, Sandra Silva

**Affiliations:** 1 RISE-Health, MAIEC Lab, Nursing School, University of Porto, Rua Dr. António Bernardino de Almeida 830/844/856, 4200-072 Porto, Portugal; 2 São Miguel Island Health Unit, Grotinha nº1, 9500-354 Ponta Delgada, Portugal

**Keywords:** epidemiological surveillance of nursing diagnoses, community health nursing, public health nursing, MAIEC, community empowerment, nursing diagnoses, primary healthcare, health information systems, ICNP^®^

## Abstract

**Highlights:**

**Public health relevance—How does this work relate to a public health issue?**

- This study identifies structural, organizational, and training conditions required to implement Epidemiological Surveillance of Nursing Diagnoses (ESND), addressing the underrepresentation of nursing-sensitive phenomena in epidemiological systems.
- It focuses on three priority public health challenges in the Azores—tobacco use, drug dependence, and adolescent decision-making—linking nursing documentation to population health needs.

**Public health significance—Why is this work of significance to public health?**

- This work reaffirms the development of a postmodern epidemiology, oriented not toward diseases, but toward Nursing Diagnoses through their epidemiological surveillance.
- Using the MAIEC model, the study reveals weak community leadership, limited participation, and insufficient coping capacity affecting ESND implementation.

**Public health implications—What are the key implications or messages for practitioners, policy makers and/or researchers in public health?**

- Findings highlight the importance of strengthening leadership, communication, and training to support nurse-centered epidemiological surveillance.
- ESND has the potential to enhance the visibility of nursing contributions and improve population health monitoring.

**Abstract:**

This study assessed community process and empowerment in a Primary Healthcare Island Unit in the Azores to support the implementation of Epidemiological Surveillance of Nursing Diagnoses (ESND), focusing on three priority areas: tobacco use, drug dependence, and adolescent decision-making related to sexuality and life planning. Strengthening the visibility of nursing-sensitive phenomena requires integrating nursing diagnoses into epidemiological surveillance systems. A multimethod descriptive study was conducted between September and November 2025, combining document analysis, a community empowerment assessment, and a structured questionnaire. The total population included 328 nurses, with 172 participants (response rate: 52.4%) using a non-probabilistic sampling approach. Data were analyzed using descriptive statistics (frequencies, percentages, means, and standard deviations). Priority nursing foci were identified according to the ICNP^®^ 2019 classification: tobacco use, drug dependence, and decision-making process related to sexuality and life planning. Results showed that all three dimensions of the MAIEC were weak: community leadership was limited, particularly in knowledge indicators; participation was constrained by unclear organizational structures and insufficient communication; and coping capacity was insufficient due to limited training and experience. Empowerment assessment confirmed structural weaknesses in leadership, organizational support, and resource mobilization. Overall, the community process and empowerment profile indicate that the conditions required to sustain ESND are not yet sufficiently developed. Strengthening leadership, improving communication, and expanding training in ESND and ICNP^®^ documentation are essential to support nurse-centered surveillance and enhance the visibility of nursing contributions to population health.

## 1. Introduction

Epidemiological Surveillance of Nursing Diagnoses (ESND) is an emerging approach in public and community health that expands traditional epidemiology beyond diseases to include nursing-sensitive phenomena such as behaviors, vulnerabilities, strengths, and decision-making patterns [1,2,3,4]. These aspects are central to prevention and early intervention but are often underrepresented in conventional surveillance systems.

A key concept underlying ESND is the nursing diagnosis. A nursing diagnosis is a clinical judgment concerning a human response to health conditions or life processes, or a susceptibility to that response [2,5]. Unlike medical diagnoses, which identify diseases or pathological conditions, nursing diagnoses focus on how individuals, families, or communities experience and respond to health-related situations [2,5].

This distinction is essential. While medical diagnoses are centered on disease identification and treatment, nursing diagnoses address human responses such as behaviors, coping patterns, adherence to treatment, risk conditions, and readiness for health improvement [2,5]. For example, while a medical diagnosis may identify a disease such as chronic respiratory illness, a nursing diagnosis may identify ineffective health management or risk behaviors that influence disease outcomes.

Nursing diagnoses are derived from systematic assessment and are directly linked to nursing interventions that aim to modify these responses [5]. These interventions are distinct in that they focus on improving individuals’ or communities’ capacity to manage health, make informed decisions, and adopt healthier behaviors, rather than treating the underlying disease itself.

This functional link between diagnosis and intervention allows nursing data to generate meaningful information about health behaviors, vulnerabilities, and social determinants [5,6,7]. As a result, integrating nursing diagnoses into epidemiological surveillance systems makes it possible to monitor dimensions of health that are often invisible in disease-centered models, thereby strengthening prevention and community-based health planning.

Integrating nursing diagnoses into epidemiological systems allows routine clinical documentation to be transformed into structured population-level data, improving the monitoring of psychosocial risks, health behaviors, and social determinants of health [5,6,7].

The theoretical and operational foundation of this study is the Community Assessment, Intervention and Empowerment Model (MAIEC), which conceptualizes the community as the unit of care and structures community nursing diagnosis around the focus of Community Management. This focus is assessed through three dimensions: Community Leadership, Community Participation, and Community Coping [8,9]. These dimensions reflect how communities organize, participate in decision-making, and mobilize resources to address priority health phenomena.

In the Autonomous Region of the Azores, ESND is particularly relevant due to persistent public health challenges. Based on the Local Health Diagnosis and the International Classification for Nursing Practice-ICNP^®^, 2019, three priority foci were identified for surveillance: tobacco use, drug dependence, and ado lescent decision-making related to sexuality and life planning [10]. These areas represent key health challenges where nursing documentation and surveillance can contribute to prevention and intervention.

This study applies the MAIEC protocol in a Primary Healthcare Island Unit using a mixed-methods design that integrates document analysis, a community empowerment assessment, and a structured questionnaire completed by 172 nurses. The objective was to diagnose community process and empowerment related to ESND implementation, identify strengths and barriers across the three MAIEC dimensions, and generate evidence to support the integration of nursing-sensitive data into epidemiological surveillance.

By examining leadership, participation, and coping capacity within the nursing community, this study provides insights that may inform other health systems seeking to strengthen nurse-centered surveillance and enhance the visibility of nursing contributions to public health.

**Figure 1:**
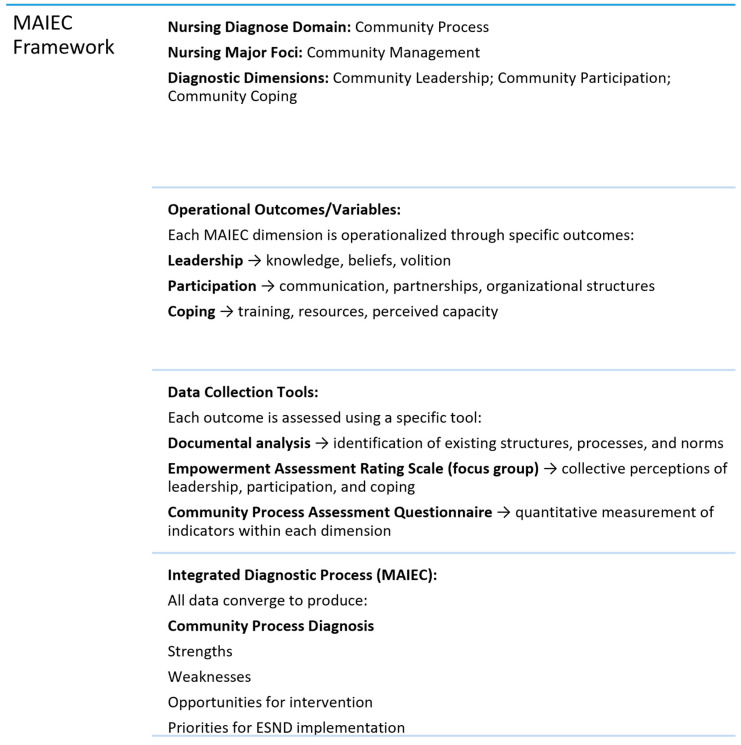
Conceptual framework linking the MAIEC, its diagnostic dimensions (Community Leadership, Community Participation, Community Coping), the operational variables assessed, and the data-collection tools used in this study (document analysis, empowerment assessment, and the Community Process Assessment Questionnaire).

To support readers’ understanding of how the MAIEC was operationalized, Figure 1 presents an integrated conceptual framework linking the model’s diagnostic dimensions, the variables assessed, and the data-collection tools used in this study.

## 2. Materials and Methods

### 2.1. Study Design

This study followed a multimethod descriptive design, consistent with the Community Assessment, Intervention and Empowerment Model (MAIEC) [8,10]. Although cross-sectional in nature, the MAIEC protocol integrates complementary data sources to support a comprehensive community nursing diagnosis. Three methodological components were combined:

Document analysis, used to identify priority nursing foci for ESND based on the Local Health Diagnosis and expert consultation;

A community empowerment assessment, conducted through a focus group using the Community Empowerment Assessment Scale [11];

A structured questionnaire, developed to operationalize the MAIEC diagnostic dimensions—Community Leadership, Community Participation, and Community Coping—and quantify indicators across the nursing workforce [8,9].

These components were analytically integrated following the MAIEC diagnostic logic: document analysis informed the selection of epidemiological priorities; the empowerment assessment contextualized community process and empowerment; and questionnaire data provided systematic measurement of these dimensions.

### 2.2. Setting

The study was conducted in a Primary Healthcare Island Unit comprising six Primary Care Centers in the Autonomous Region of the Azores, Portugal. The island presents specific demographic and epidemiological challenges, including high tobacco-related morbidity, increasing drug dependence, and elevated adolescent pregnancy rates, which informed the selection of priority foci for ESND [10].

### 2.3. Participants

The target population consisted of 328 nurses working in the Island Health Unit. A total of 172 nurses completed the questionnaire (response rate: 52.4%), representing all six primary care centers and including generalist nurses, specialist nurses, and nurse managers.

A focus group was conducted on 18 September 2025, involving eight nurses, each representing one primary care extension of the six health centers, to ensure representation across the Island Health Unit for the community empowerment assessment [11]. All nurses in the Island Health Unit were eligible to participate, and no additional screening criteria were applied.

### 2.4. Instruments

#### 2.4.1. Document Analysis

An in-depth analysis of the Local Health Diagnosis developed by the Public Health Unit of the Island Health Unit was conducted, followed by consultation with the same unit. Based on this process, and in accordance with the International Classification for Nursing Practice (ICNP^®^ 2019) [10], three priority nursing foci were identified for ESND: tobacco use, drug dependence, and decision-making processes related to sexuality and life planning. These foci reflect key community health challenges and align with the MAIEC diagnostic logic for identifying priority nursing-sensitive phenomena [8,9].

#### 2.4.2. Community Empowerment Assessment Scale

Community empowerment was assessed using the Community Empowerment Assessment Scale [9], the Portuguese adaptation of the Empowerment Assessment Rating Scale developed by Laverack [9]. The scale evaluates nine domains: community participation, ability to assess the problem, local leadership, organizational structures, resource mobilization, links to others, ability to “ask why”, program management, and relationship with external agents, scored from 1 (low empowerment) to 5 (high empowerment) [9,11]. During the focus group, participants scored each domain individually, followed by a structured consensus discussion to establish the final collective score [11].

These domains are operationalized according to Laverack’s framework. For example, community participation refers to the extent to which community members are actively involved in identifying problems, engaging in decision-making processes, and engaging in collective actions. Each domain represents a structural dimension of community empowerment relevant to the development and sustainability of health initiatives.

The focus group followed a structured rating and consensus procedure in accordance with the Community Empowerment Assessment Scale protocol, rather than a qualitative thematic analysis, as its purpose was to generate collective scoring across predefined empowerment domains.

#### 2.4.3. Community Process Assessment Questionnaire

The questionnaire was developed based on the operational definitions of the three MAIEC diagnostic dimensions—Community Leadership, Community Participation, and Community Coping [8,9]. It included sociodemographic and professional variables and 27 structured items assessing knowledge, beliefs, volition, communication, organizational structures, partnerships, training, experience, and perceived capacity.

Items were scored using ordinal scales (0–4 or 0–5). Content validity was established through expert review by three specialists in Community Health Nursing and MAIEC methodology [8,9]. All instruments were administered in Portuguese. Internal consistency was assessed using Cronbach’s alpha, yielding acceptable reliability across all three diagnostic dimensions.

### 2.5. Procedures

#### 2.5.1. Identification of Priority Nursing Foci

Priority nursing foci were identified through document analysis and expert consultation, in accordance with the ICNP^®^ 2019 classification and the MAIEC protocol [8,9,10].

#### 2.5.2. Data Collection

Data collection was conducted between 1 September and 30 November 2025. The questionnaire was distributed online through institutional channels, ensuring access was restricted to the target population.

The community empowerment focus group was conducted in September 2025, following the structured procedures of the MAIEC protocol [8,9,11,12]. Focus group participants were also included in the questionnaire phase, as the procedure did not involve exposure to questionnaire items or training that could influence responses.

### 2.6. Data Analysis

Quantitative data were analyzed using IBM SPSS Statistics version 29.0 [13], applying descriptive statistics, including frequencies, percentages, means, and standard deviations. Variables were organized according to the three MAIEC diagnostic dimensions [8]:

- Community Leadership (knowledge, beliefs, volition);
- Community Participation (communication, partnerships, organizational structures);
- Community Coping (experience, training, perceived capacity).

This analytical approach is consistent with the diagnostic purpose of the study, which aimed to characterize community process and empowerment rather than test causal relationships [8,10].

Focus group data were analyzed using the structured consensus procedure defined by the Community Empowerment Assessment Scale [9,11].

### 2.7. Sample Precision and Power Considerations

Sample adequacy was evaluated using precision parameters. The margin of error was 5.4% at a 95% confidence level, based on a conservative expected proportion of 50%.

### 2.8. Ethical Considerations

The study was conducted in accordance with the Declaration of Helsinki and national and institutional guidelines for research involving human participants. Ethical approval was granted by the Ethics Committee of the Island Health Unit (approval number: USISMG/2024/14057). Participation was voluntary, informed consent was obtained from all participants, and all data were anonymized and securely stored.

## 3. Results

This section presents the findings according to the three diagnostic dimensions of the MAIEC—Community Leadership, Community Participation, and Community Coping—highlighting the key patterns that characterize the community process related to ESND implementation. Numerical details are provided in the corresponding tables and figures to avoid redundancy in the narrative. Overall, the results show that leadership, participation, and coping capacity were insufficient to support ESND implementation.

### 3.1. Sample Characteristics

A total of 172 nurses participated in the study (response rate: 52.4%). The sample was predominantly female, professionally experienced, and largely composed of nurses with more than ten years of service in the Island Health Unit. Most participants worked in direct care, and all eight primary care extensions were represented. Table 1 summarizes the sociodemographic and professional characteristics.

Internal consistency of the Community Process Assessment Questionnaire was acceptable to good across all three diagnostic dimensions (Cronbach’s α = 0.79–0.86), as shown in Table 2.

### 3.2. Community Process Diagnosis

#### 3.2.1. Overview

Across the three MAIEC dimensions, the results indicate weak community leadership, limited community participation, and insufficient community coping. Structural constraints, limited training, and low organizational visibility emerged as cross-cutting barriers.

#### 3.2.2. Community Leadership

Most nurses reported low familiarity with key documents, including the Local Health Diagnosis (61.6%) and the Local Health Plan (52.3%), with few reporting having read them.

**Figure 2:**
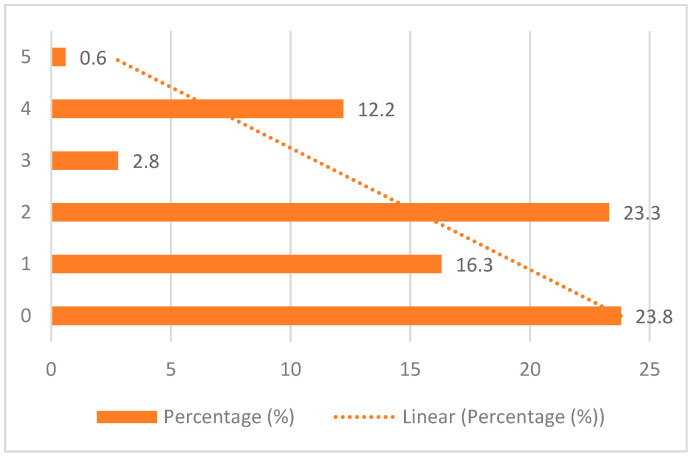
Distribution of Knowledge Levels Regarding ESND (*N* = 172).

Knowledge about documentation in the three ICNP^®^ priority foci—tobacco use, drug dependence, and decision-making process—was also low to moderate (Figure 2 and Table 3 and Table 4).

- Beliefs

**Figure 3:**
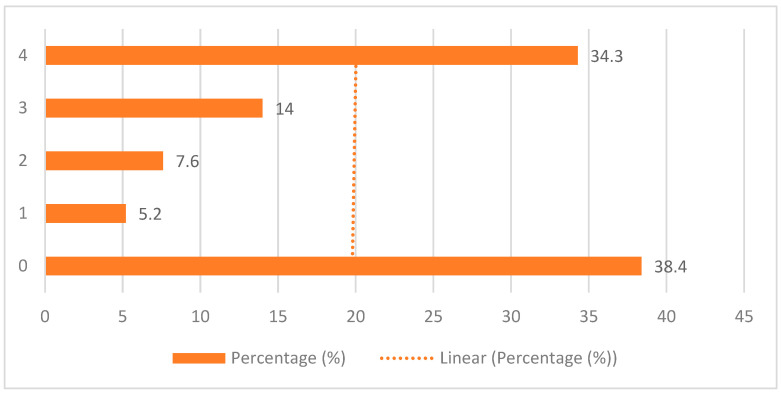
Distribution of Belief Levels Regarding the Relevance of ESND (*N* = 172).

Beliefs about ESND showed a mixed pattern (Figure 3). While many nurses recognized its potential value, uncertainty and ambivalence were also evident.

Across the Community Leadership dimension, these findings suggest that although conceptual understanding remains limited, there is openness to integrating ESND into clinical practice. This openness represents an important enabling factor that may facilitate future capacity-building efforts.

- Volition

**Figure 4:**
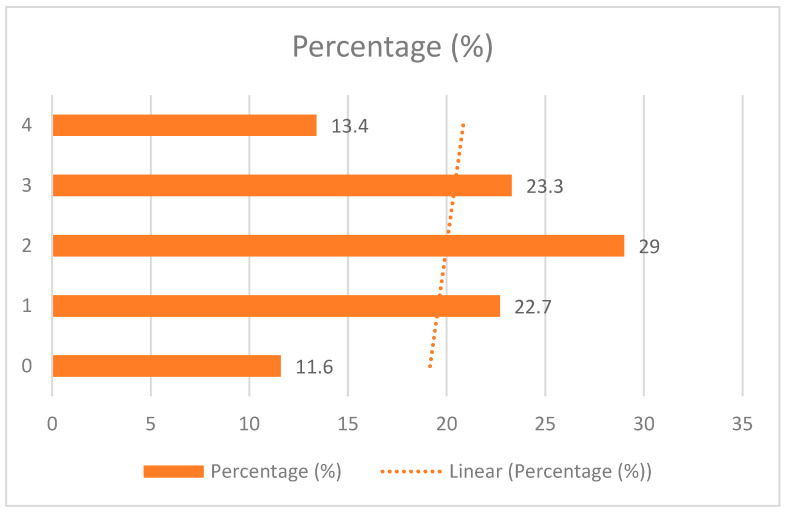
Distribution of Volition Indicators Regarding ESND (*N* = 172).

Volition indicators showed moderate willingness to engage in ESND-related activities (Figure 4).

### 3.3. Community Participation

Community participation was limited, particularly in relation to organizational structures, communication pathways, and leadership support.

Indicators related to the clarity of the ESND process, communication mechanisms, and partnerships (Table 5) revealed fragmented organizational support. Many nurses reported uncertainty regarding the existence of organizational structures to support ESND (Table 6).

**Figure 5:**
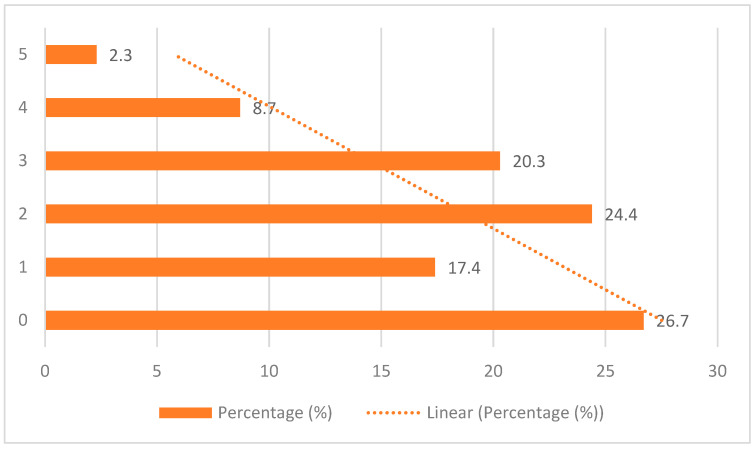
Distribution of Perceived Clarity of the ESND Process (communication-related) (*N* = 172).

Communication about ESND-related activities was perceived as insufficient (Figure 5). Perceptions of leadership support were also limited, indicating a lack of clear coordination for ESND implementation (Table 7).

### Community Coping

Community coping capacity was insufficient, with limited training, reduced experience, and low perceived capacity to engage in ESND-related activities (Table 8).

Indicators related to training and experience showed that most nurses had little or no prior exposure to ESND or similar surveillance processes. Perceived strengths within the community were moderate, although many participants reported limitations in skills and resources required for ESND implementation (Table 8).

### 3.4. Integrated Diagnostic Interpretation

When analyzed collectively, the three MAIEC dimensions indicate that the conditions required to sustain ESND are not yet sufficiently developed. The diagnostic profile is characterized by:

- Low knowledge but moderate volition (Community Leadership).
- Limited organizational clarity and communication (Community Participation).
- Insufficient training and perceived capacity (Community Coping).

These findings identify priority areas for intervention within the MAIEC framework.

### 3.5. Community Empowerment Assessment

**Figure 6:**
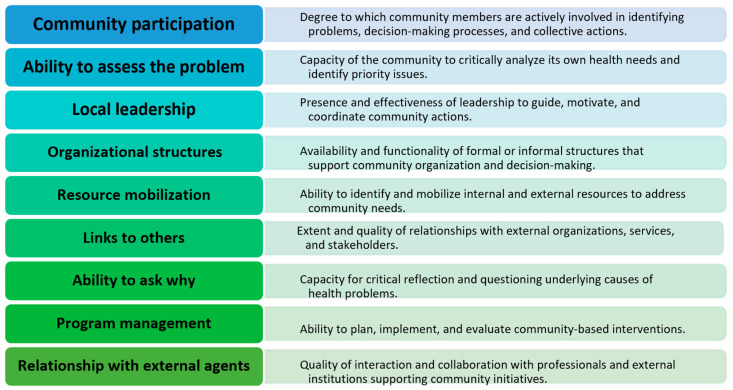
Operational description of the nine domains of the Community Empowerment Assessment Scale [9,11].

The community empowerment assessment, conducted on 18 September 2025, generated a multidimensional profile across the nine domains of the Portuguese version of the Community Empowerment Assessment Rating Scale [11], originally developed by Laverack [9]. To facilitate understanding of the nine domains, Figure 6 presents a concise description of each domain.

**Figure 7:**
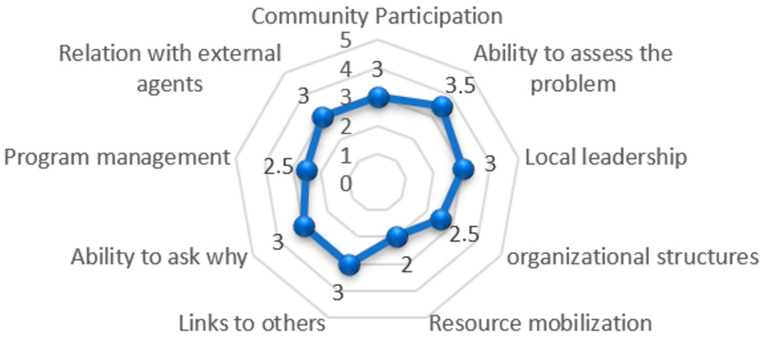
Community Empowerment Profile Across the Nine Domains of the Community Empowerment Assessment Rating Scale (Portuguese version) [11].

All of these domains reflect structural components of empowerment as defined by Laverack [9]. Figure 7 presents a radar chart summarizing the empowerment scores, providing an integrated view of the nursing community’s capacity to engage in ESND-related processes.

The radar chart displays mean values for each domain. To complement this visualization, Table 9 reports the corresponding mean and standard deviation for each domain.

The results indicate a moderate level of community empowerment, characterized by strengths in motivation and willingness to engage (volition), but limited knowledge, organizational structures, and coping resources. This profile reflects the presence of foundational elements of empowerment, although these are not yet sufficiently consolidated to support sustained and autonomous ESND implementation.

Variability across services suggests differences in organizational support, communication processes, and access to training.

Overall, the empowerment profile reflects a community with developmental potential, but with insufficient structural and organizational conditions to support sustained ESND implementation.

## 4. Discussion

This study examined community process and empowerment in a Primary Healthcare Island Unit to support the implementation of Epidemiological Surveillance of Nursing Diagnoses (ESND). The findings indicate weak community leadership, limited participation, and insufficient coping capacity, reflecting constrained organizational conditions for integrating nursing-sensitive data into epidemiological surveillance.

### 4.1. Community Leadership: Knowledge Gaps and Motivational Potential

The results highlight important gaps in knowledge, particularly regarding epidemiological documents and documentation practices. Limited familiarity with the Local Health Diagnosis and Local Health Plan reduces the ability of nurses to engage in systematic surveillance processes. At the same time, the presence of moderate levels of volition indicates a willingness among professionals to engage in ESND-related activities.

This combination of low knowledge and moderate motivation suggests that the nursing community possesses important enabling elements for ESND but lacks the structural and educational support required to translate motivation into effective surveillance practices.

### 4.2. Community Participation: Organizational Fragmentation and Limited Communication

Community participation indicators revealed limited clarity about ESND processes, insufficient communication pathways, and weak leadership support. These findings are consistent with the MAIEC, which emphasizes that effective community management depends on clear organizational structures, shared decision-making, and coordinated leadership [8,9]. The absence of these conditions in the Island Health Unit constrains the implementation of ESND and limits collective engagement among professionals. Strengthening organizational alignment, clarifying leadership roles, and improving communication processes are essential to support ESND implementation.

### 4.3. Community Coping: Training Needs and Perceived Capacity

Low levels of training and experience related to ESND indicate that the nursing community lacks the competencies required to document and monitor nursing-sensitive phenomena systematically. These findings are consistent with previous research showing that integrating nursing diagnoses into surveillance systems depends on sustained professional development and opportunities for experiential learning [5,6,7]. The limited perceived capacity identified in this study highlights the need for targeted training, resource allocation, and supportive supervision to strengthen community coping and support ESND implementation.

### 4.4. Community Empowerment

Empowerment assessment complements the diagnostic findings by revealing structural limitations in leadership, organizational structures, and resource mobilization—domains that are central to community empowerment according to Laverack’s framework [9,11]. These findings reinforce that the nursing community’s empowerment is constrained, particularly in structural and organizational dimensions. Strengthening these domains is essential to support sustained engagement in ESND-related processes. These results are consistent with the MAIEC, which conceptualizes empowerment as a structural condition for effective community management [8,9].

### 4.5. Implications for Health Systems Integrating Nursing-Sensitive Data

The findings of this study provide insights that may be relevant for health systems seeking to integrate nursing-sensitive data into epidemiological surveillance. Specifically, the results indicate that ESND implementation depends on the following conditions:

- Leadership structures that provide clear direction and accountability;
- Engagement and participation mechanisms that promote shared understanding and collaboration;
- Training and professional development to build competencies in documentation and surveillance;
- Organizational support to ensure communication, visibility, and resource allocation;
- Clear priorities that align nursing documentation with community health needs.

These conditions are not specific to the Azores context but represent foundational elements for health systems aiming to strengthen nursing contributions to population health monitoring.

Mixed-method approaches, such as the one used in this study, can support the identification of both structural and cultural conditions required to build sustainable, nurse-centered surveillance processes.

### 4.6. Limitations of the Study

This study has several limitations. Its cross-sectional design does not allow causal inferences regarding the relationships between community empowerment, community process indicators, and ESND implementation. Data were collected through self-reported measures, which may be subject to recall and social desirability bias. Although the response rate was acceptable, non-response bias cannot be excluded, particularly among nurses less engaged in organizational processes. Selection bias is also possible, as nurses with greater interest in ESND may have been more likely to participate. The study was conducted in a single Island Health Unit in the Autonomous Region of the Azores, which may limit the transferability of findings to settings with different organizational structures or epidemiological profiles. Finally, the community empowerment assessment was based on a focus group with representatives from each primary care extension; although consistent with the MAIEC protocol, this approach may not fully capture the diversity of perspectives within the broader nursing community.

This study focused on identifying structural conditions within the MAIEC framework rather than assessing attitudinal gradients. Accordingly, dichotomous (yes/no) items were considered appropriate for the diagnostic purpose of the study. Future research using Likert-type scales may provide a more detailed understanding of nurses’ perceptions regarding ESND implementation.

### 4.7. Contributions to Clinical Practice

This study provides insights into strengthening clinical practice in primary healthcare. The identification of knowledge and training gaps highlights the need for structured educational programs on ESND and ICNP^®^ documentation, which may enhance diagnostic accuracy and improve the visibility of nursing-sensitive phenomena. Improving communication processes and clarifying leadership roles may support more consistent documentation practices and promote shared responsibility for epidemiological surveillance. These findings align with international recommendations for improving the quality and interoperability of nursing documentation [6,14].

### 4.8. Contributions to Research

This study advances research in community health nursing by operationalizing the MAIEC in the context of ESND [8,9]. The diagnostic findings provide a baseline for future intervention studies aimed at strengthening community empowerment and evaluating the impact of ESND implementation on health outcomes. The study also highlights opportunities for research on the integration of nursing diagnoses into health information systems and on the development of digital tools to support real-time surveillance, consistent with global trends in nursing informatics [14,15,16].

### 4.9. Contributions to Society

At a societal level, this study highlights the role of nursing as a key contributor to public health surveillance. By identifying barriers and strengths within the nursing community, the findings support the development of more responsive, equitable, and community-centered health systems. Strengthening ESND can enhance the monitoring of health behaviors, vulnerabilities, and decision-making processes, contributing to more informed public health policies and improved population health in the Autonomous Region of the Azores.

## 5. Conclusions

This study shows that the implementation of Epidemiological Surveillance of Nursing Diagnoses (ESND) depends on community process and empowerment rather than conceptual relevance alone.

In the studied Primary Healthcare Island Unit, leadership, participation, and coping capacity were insufficient to support the integration of nursing-sensitive data into epidemiological surveillance.

Strengthening leadership, improving communication and organizational alignment, and expanding training in ESND and ICNP^®^ documentation are essential to support nurse-centered surveillance.

These findings highlight key conditions—leadership, engagement, training, and organizational support—that are relevant for health systems seeking to strengthen population health monitoring through nursing contributions.

## Figures and Tables

**Table 1:** Sociodemographic and professional characteristics of the nursing community (*N* = 172).

Variable	Category	Frequency	Percentage (%)
Gender	Female	154	89.5
	Male	17	9.9
	Prefer not to answer	1	0.6
Age	20–30	7	4.1
	31–40	43	25.0
	41–50	90	52.3
	>51	32	18.6
Years of Service (Island Unit)		3	1.7
	1–5 years	30	17.4
	6–10 years	17	9.9
	>10 years	122	70.9
Area of Practice	Direct Care	145	84.3
	Management	27	15.7
Professional Category	Generalist Nurse	92	53.5
	Specialist Nurse	74	43.0
	Nurse Manager	6	3.5
Previous PHC Experience	Yes	93	54.1
	No	79	45.9

**Table 2:** Internal consistency of the Community Process Assessment Questionnaire (Cronbach’s alpha).

Diagnostic Dimension	Instrument Section	Cronbach’s α
Community Leadership	Knowledge, beliefs, volition	0.82
Community Participation	Communication, partnerships, organizational structures	0.79
Community Coping	Experience, training, resources, perceived capacity	0.86

**Table 3:** Distribution of the Knowledge data regarding Community Leadership Dimension of the MAIEC (*N* = 172).

Dimension	Category/Score	Frequency	Percentage (%)
Knowledge: Local Health Diagnosis	No	106	61.6
	Heard of it, never read	51	29.7
	Yes	15	8.7
Knowledge: Local Health Plan	No	90	52.3
	Heard of it, never read	61	35.5
	Yes	21	12.2

**Table 4:** Descriptive statistics of knowledge regarding documentation in the priority ICNP^®^ nursing foci (Community Leadership dimension).

	*N*	Minimum	Maximum	Mean	Standard Deviation
Knowledge about documentation in the foci “use of tobacco”	172	0	5	1.86	1.374
Knowledge about documentation in the foci “Drug dependence”	172	0	4	1.98	1.408
Knowledge about documentation in the foci “Decision-making Process (regarding sexuality and life project”	172	0	4	1.88	1.337

**Table 5:** Indicators related to the existence of community partnerships to promote ESND (Community Participation dimension).

Dimension	Category	Frequency	Percentage (%)
Existence of Partnerships	Unknown	119	69.2
	No	24	14.0
	Yes	29	16.9

**Table 6:** Community participation indicators related to the existence of organizational structures to promote ESND.

Dimension	Category	Frequency	Percentage (%)
Perceptions on the existence of Organizational Structures to promote ESND	Unknown	102	59.3
	No	19	11.0
	Yes	51	29.7

**Table 7:** Community participation indicators related to the existence of formal leadership to promote ESND.

Dimension	Category	Frequency	Percentage (%)
Existence of formal Leadership to promote ESND	No	94	54.7
	Yes	78	45.3

**Table 8:** Community coping indicators related to experiences, training and strengths to promote ESND.

Dimension	Category	Frequency	Percentage (%)
Previous ESND Experience	No	147	85.5
	Yes	25	14.5
Training: Tobacco Use Documentation	No	140	81.4
	Yes	32	18.6
Training: Drug Dependence Documentation	No	146	84.9
	Yes	26	15.1
Training: Decision-Making Process (sexuality and life project) Documentation	No	152	88.4
	Yes	20	11.6
Training: ICNP^®^ Nursing Diagnosis	No	74	43.0
	Yes	98	57.0
Perceived Strengths for ESND Project	No	80	46.5
	Yes	92	53.5

**Table 9:** Mean and standard deviation for each domain represented in the radar chart.

Domain	Mean	SD
Community Participation	3.0	0.53
Ability to assess the problem	3.5	0.76
Local leadership	3.0	0.71
Organizational structures	2.5	0.64
Resource mobilization	2.0	0.58
Links to others	3.0	0.67
Ability to ask why	3.0	0.60
Program management	2.5	0.72
Relation with external agents	3.0	0.69

## Data Availability

The original contributions presented in this study are included in the article. Further inquiries can be directed to the corresponding author.
